# Parenchymal Organ Changes in Two Female Patients With Cornelia de Lange Syndrome: Autopsy Case Report

**DOI:** 10.7759/cureus.9767

**Published:** 2020-08-15

**Authors:** Martin Mangelov, Niya Balgarinova, Krassimira Zaykova, George S Stoyanov, Deyan L Dzhenkov

**Affiliations:** 1 Faculty of Medicine, Medical University - Varna, Varna, BGR; 2 General and Clinical Pathology/Forensic Medicine and Deontology, Medical University - Varna, Varna, BGR

**Keywords:** cornelia de lange syndrome, pathology, autopsy, pyramidal organs

## Abstract

Cornelia de Lange (CdLS) is a rare congenital disorder with multifactor etiology. The syndrome features a wide variety of physical and cognitive hallmarks such as distinctive facial appearance, small stature, bone, and gastrointestinal abnormalities. Two cases of patients clinically diagnosed with CdLS are reported. Both cases were diagnosed and treated at the St. Marina University Hospital, Varna, Bulgaria and were referred for autopsy after death. The first case was of a female patient, aged 7 and the second of a 17-years-old female. Both patients had a family history, severe features, and complications associated with CdLS. In both cases, the liver had normal anatomical proportions with a slightly flattened shape especially pronounced in the second case where the liver had a pyramidal shape with sharp edges. The kidneys in both patients were with a flattened pyramidal shape, with the tip located at the hilum and a base toward the lateral abdominal side. Both patients also had a pyramidal shaped spleen, again with the tip located at the hilum, with the second patient having multiple accessory spleens along the splenic artery. The cause of death in both patients was determined as complications from CdLS. The pyramidal form of the parenchymal organs is a manifestation that has so far not been described in CdLS patients. Despite atrophic organs sometimes having the same appearance, different organs are rarely affected identically, hence these changes can be considered as specific features of CdLS.

## Introduction

Cornelia de Lange syndrome (CdLS) also known as Amsterdam dwarfism is a rare developmental genetic disorder with an incidence of 1.23 per 100,000 live births and a complex variety of genetic and clinical features [1-4]. The syndrome includes a wide variety of physical and cognitive hallmarks ranging from mild to severe [2-4].

The most common features of CdLS include distinctive facial appearance, small staturе, mental retardation with physical growth delay, bone abnormalities, and those involving multiple body organs, most often gastrointestinal abnormalities [2-8]. The distinctive facial features represent thick eyebrows, a small upturned nose, long or smooth philtrum, and a thin upper lip. The diagnosis of CdLS is primarily based on these clinical findings [3-5].

Most children with CdLS are clinically diagnosed after birth or in childhood based on a comprehensive clinical evaluation and recognition of the characteristic morphological findings [2-5]. A diagnosis of CdLS should be considered in children who exhibit certain distinctive facial features. If the signs and physical features associated with the condition are very mild, diagnosis can be challenging [2-4]. Life expectancy in CdLS is generally favorable, with some patients living well into adulthood [2,6-7].

## Case presentation

We report two cases of female children clinically diagnosed with CdLS with many external deformities and multiple organ involvement. They had no family connection, but they both had a family history of pathological pregnancies and relatives who had CdLS. They have been diagnosed and treated at the St. Marina University Hospital, Varna, Bulgaria.

Medical history of the first case

The first patient was a seven-year-old female born by natural means after a normal pregnancy weighing 2800g. She exhibited all signs of CdLS and had a medical history of frequent hospitalizations and a family history of a sister deceased at six months of age, who also had CdLS.

Due to several surgical interventions and perforation of the esophagus, a gastrostomy was placed. The patient was admitted to the Department of Pediatric Diseases, St. Marina University Hospital with complaints of vomiting and leakage of a dark brownish liquid from the gastrostomy after meals.

Present complaints included diaphragmatic hernia and esophagus stenosis, chronic gastric ulcer, ascites, bronchiectasis disease with acute bronchitis and bronchiolitis, focal atelectasis, emphysema, and an autonomous thrombus in a branch of the pulmonary artery, right-sided pleural fibrosis, pericardial effusion.

Medical history of the second case

The second patient is a 17-year-old female born from a seventh pathological pregnancy, that presented with neurological symptoms in the early neonatal period. She had a history of frequent hospitalizations due to multiple neurological symptomatic, measles infection, atopical dermatitis, and a fracture of the femur. The reason for the current hospitalization was a deteriorated overall condition, fever lasting for two days, lack of appetite, difficulty in breathing, and restlessness. After hospitalization, the symptomatology progresses with the appearance of respiratory failure, upper and lower dyspeptic syndrome, and hemodynamic failure. Despite the performed medical and resuscitation measures, the organ failure progresses and *exitus letalis* was registered.

After death, both patients were referred for autopsy, with several unusual similarities between the parenchymal organs in both patients being observed. Both autopsies were video documented in full.

Autopsy findings

**Figure 1:**
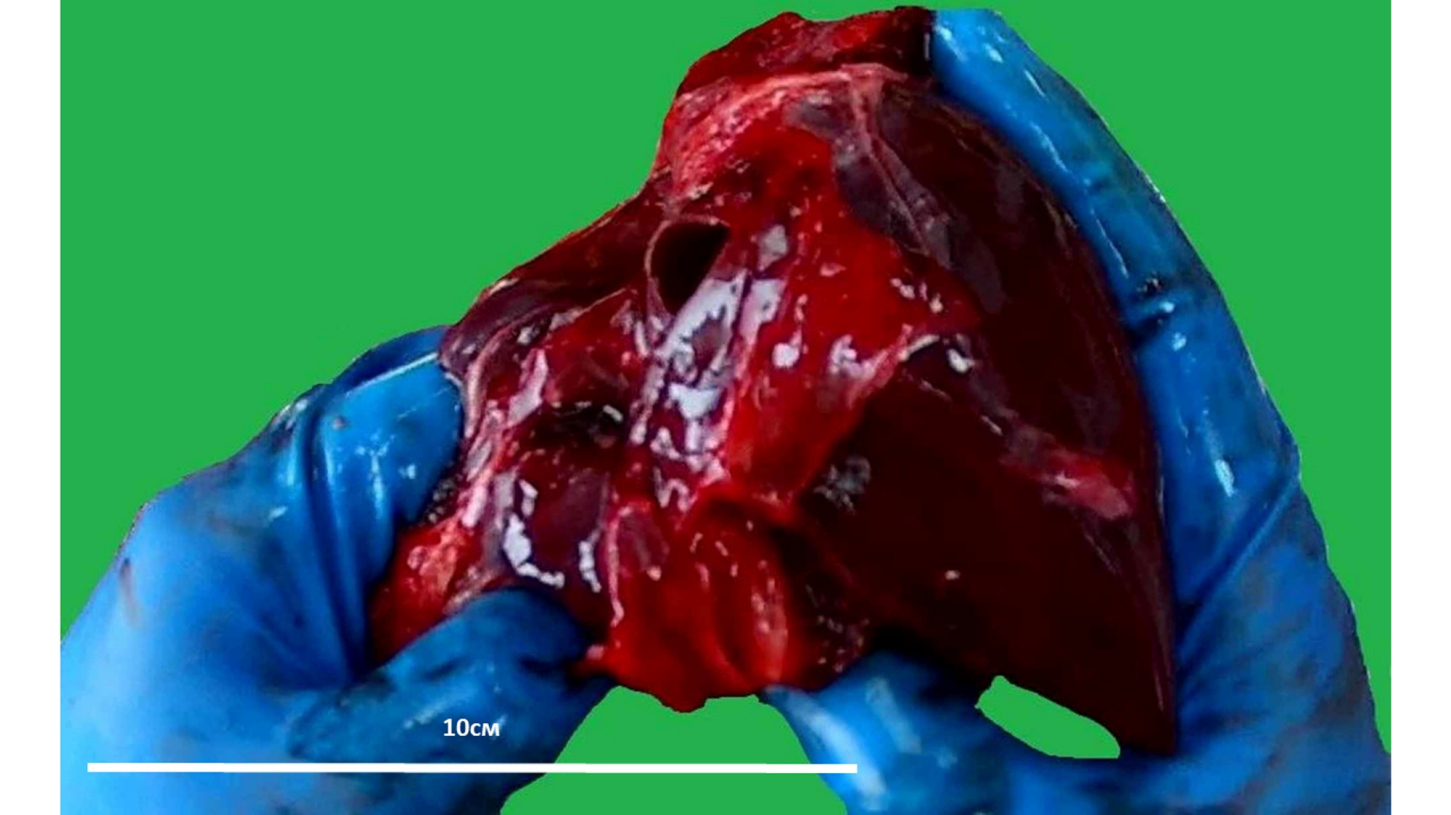
Gross structure of the liver from the first patient Organ weight 420g, age reference weight 437-1009 [8]. Note: the image is a snapshot from the autopsy video recording.

In both cases, the liver had normal anatomical proportions with a slightly flattened shape especially prominent in the second patient, where the liver had а well-defined pyramidal shape with sharp edges (Figure 1). Liver weight was normal for the age of the patients. On cross-section, the liver had a yellowish color. The gallbladder showed no morphological changes.

**Figure 2:**
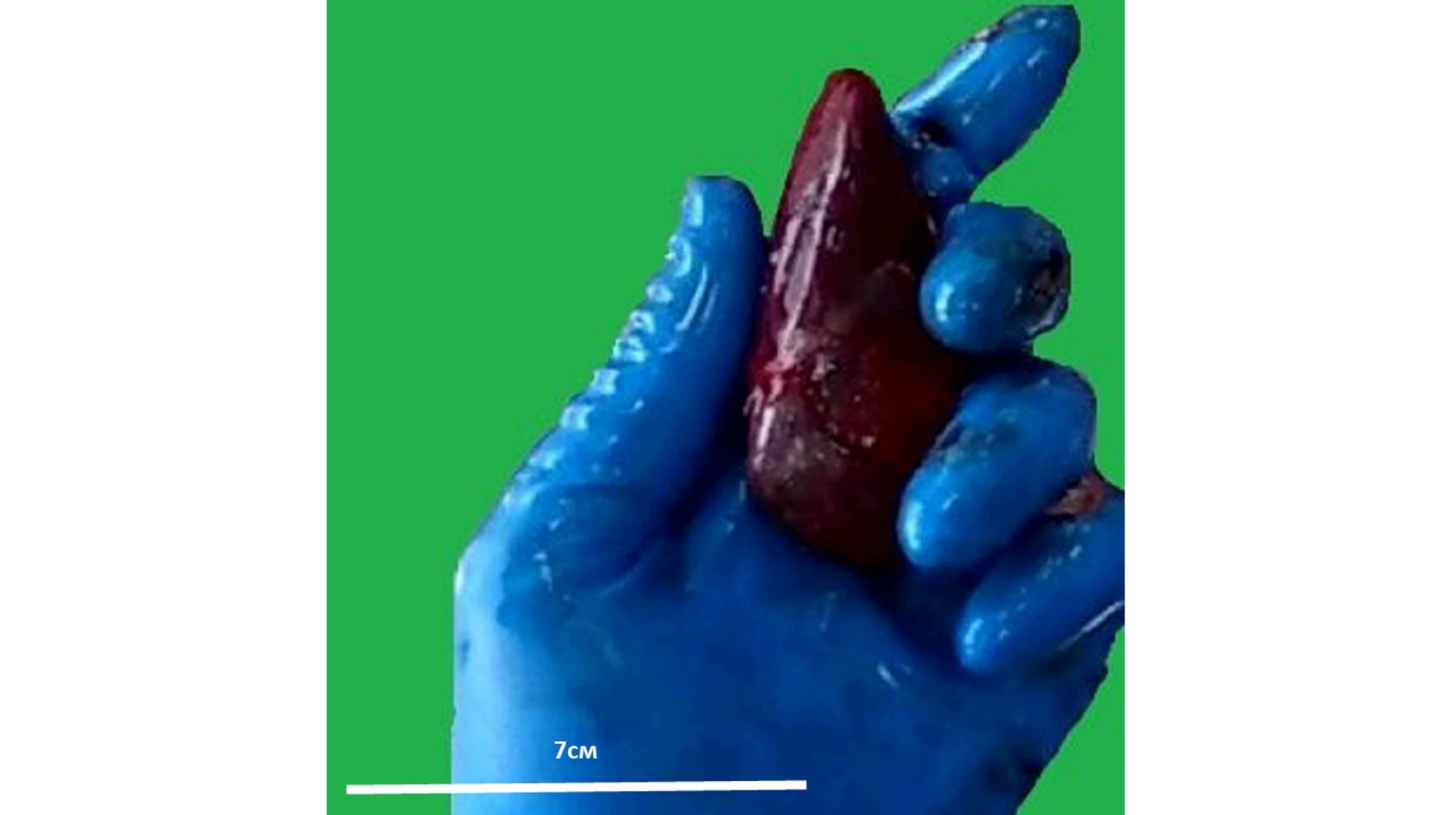
Gross structure of the kidney of the second patient Left kidney weight - 80g, right kidney weight - 70g. At age of 17 organs should be 10% less than reference for adults - 100-140g. Note: the image is a snapshot from the autopsy video recording.

Kidney weight was in the normal range for the age of both patients, with similar changes as those observed in the liver - flattened and with a pyramidal shape, the tip of which was located at the hilum and a base pointing toward the lateral abdominal wall (Figure 2). The cross-section and renal pelvis revealed no morphological changes.

**Figure 3:**
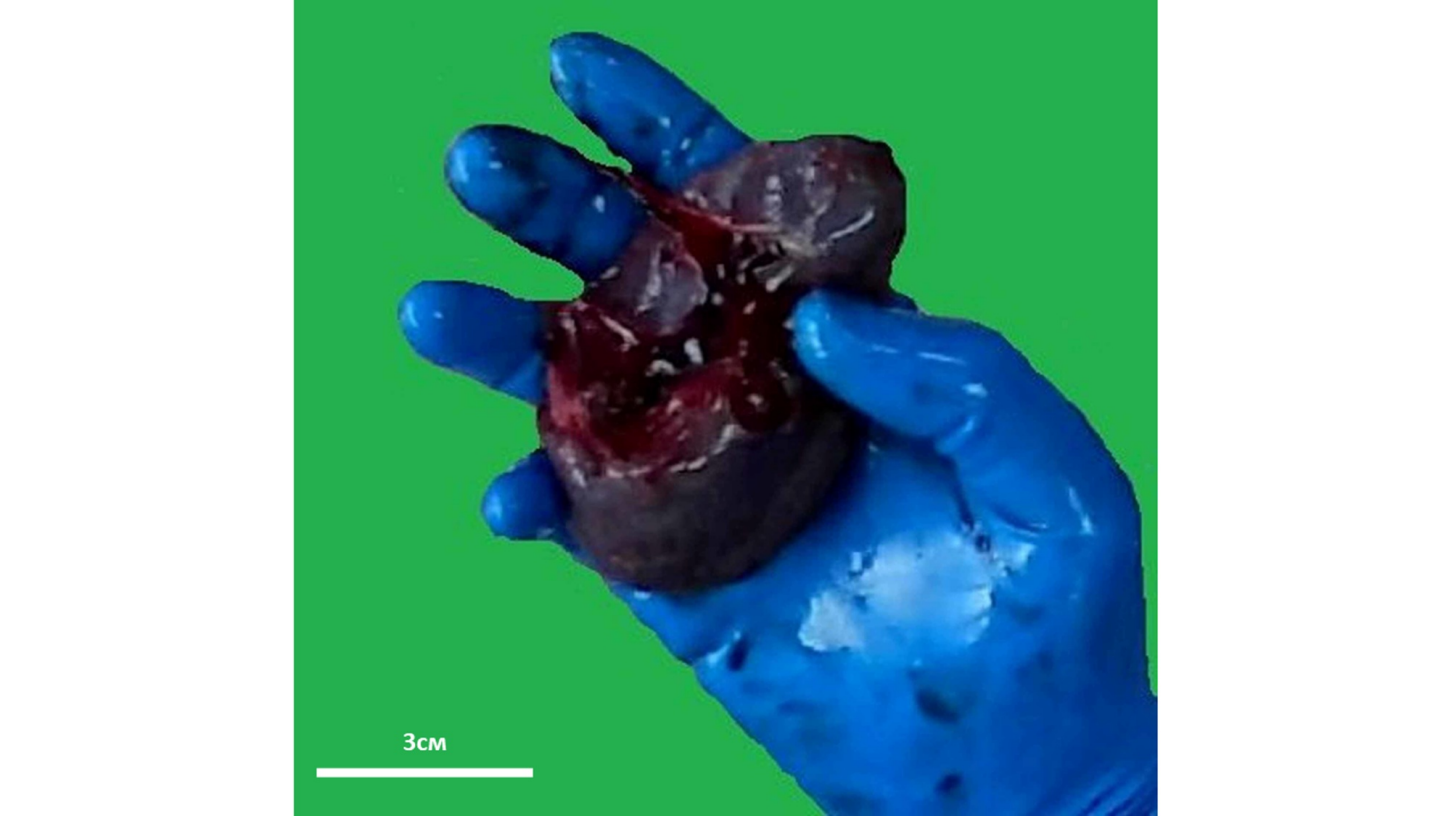
Gross structure of the spleen of the first patient Organ weight - 130g, reference weight - 16-162g [8]. Note: the image is a snapshot from the autopsy video recording.

Spleens were the third parenchymal organ that had the unusual flattened pyramidal shape and weight in the normal range for the age of the patients (Figure 3). Furthermore, the first patient had multiple small accessory spleen along the splenic lienal artery.

The liver, kidneys, and spleen in both patients showed no histological changes.

**Figure 4:**
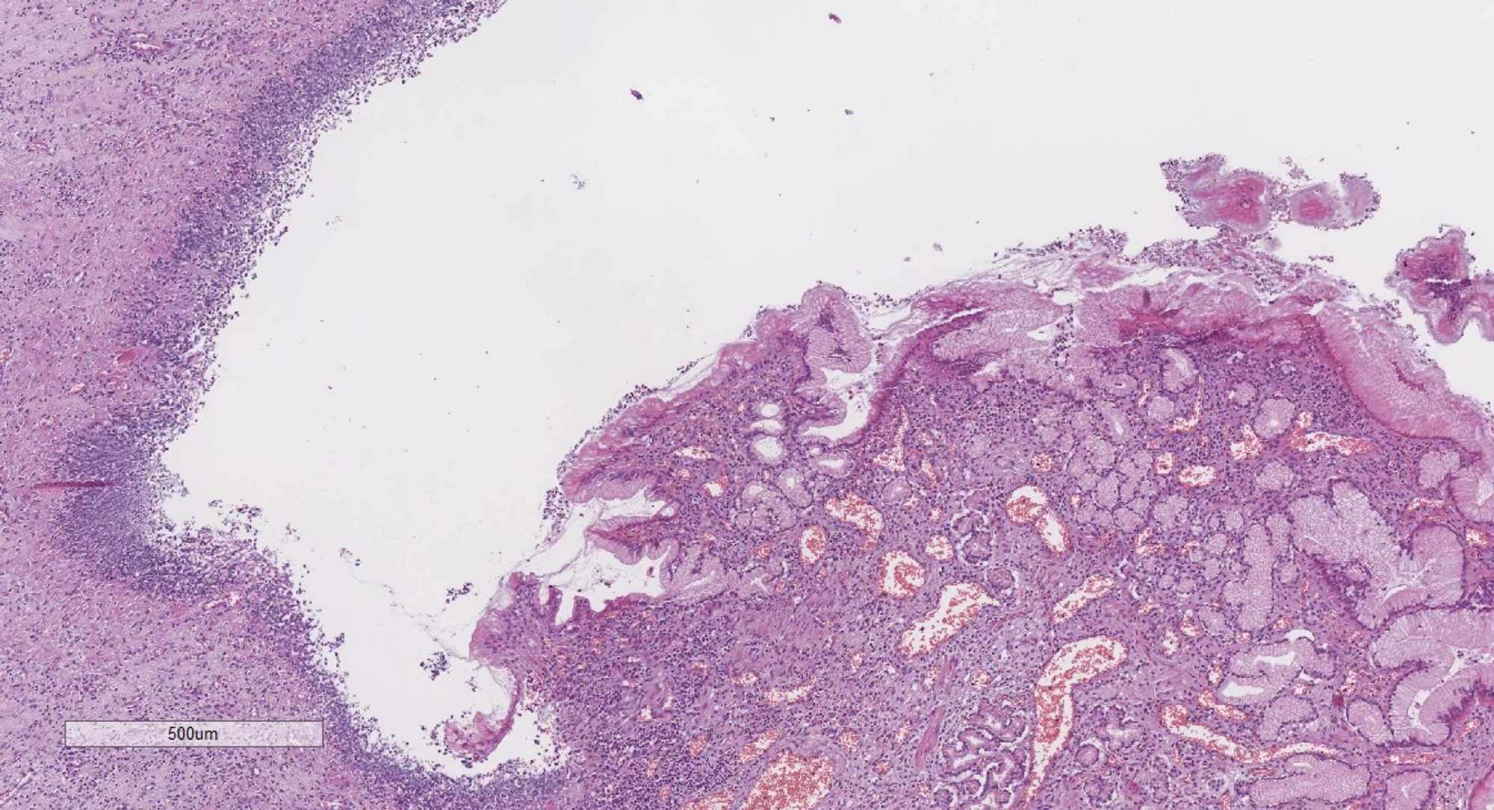
Peptic ulcer from the first patient, hematoxylin and eosin stain, original magnification x40

**Figure 5:**
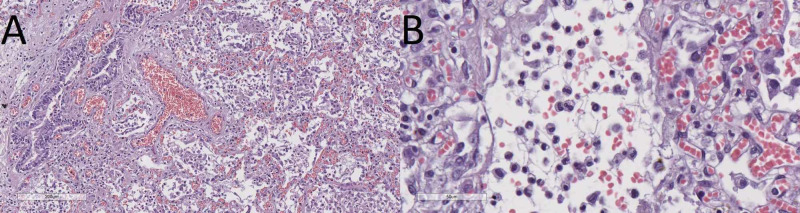
Lung histology of the second case Purulent bronchopneumonia, hematoxylin and eosin stain, original magnification x100 (A) and alveoli infiltrated by neutrophils and scant fibrin strands and erythrocytes, hematoxylin and eosin stain, original magnification x200 (B).

The cause of death in the first patient was an active bleeding stomach ulcer and bronchopneumonia in the second patient (Figures 4, 5).

## Discussion

CdLS is most often diagnosed on physical examination [2-4]. Detailed family medical history also plays an important role, with genetic tests having a disputable role in the diagnosis of the condition, as there have been multiple mutations found in patients with similar phenotypic appearance [2,6,7,9].

Prenatal diagnosis of CdLS is difficult, with an ultrasound after the 18th gestational week and family history of CdLS being the only two mechanisms of suspecting the presence of the condition [3,6,10]. The safety of ultrasonography makes it a powerful tool in the hands of gynecologists, obstetricians, and pediatricians. A thorough examination and close attention should be made to the face profile, arms, and hands, fingers, internal organ changes such as heart defects, diaphragmatic hernia, or gastrointestinal defects [2-4,6,10]. Ultrasound examination is not the perfect tool to exclude the possibility of the syndrome, but it’s a convenient way to detect severe cases [6,10].

Genetic tests do not always yield diagnostic results, as the condition is with multifactor genetic etiology and a universal genetic defect has not yet been identified [2,3,6,11].

The results we report are described for the first time, which gives us a reason to consider them as potential diagnostics criteria, which still needs further research. Enriching the list of signs and symptoms with these specific organ changes in CdLS can be of benefit to the diagnosis of borderline cases [3,5,6]. Furthermore, these changes may manifest as pathological later in life with biliary and renal pelvis obstruction, kidney stones, or proneness of the spleen to rupture.

## Conclusions

CdLS is a rare congenital disorder. Herein we report two autopsy cases of CdLS patients in different age groups with no family connection and similar changes in the parenchymal organs, with a pyramidal shape of the liver, spleen, and kidneys. These changes can be considered as a component of the syndrome, however, a larger cohort is needed to specify their role in the condition.
